# An Atypical Sonographic Presentation of an Endometrial Polyp in a Postmenopausal Woman: A Case Report

**DOI:** 10.7759/cureus.73107

**Published:** 2024-11-06

**Authors:** Xiaohong Yao, Xuandan Ye, Lixia Chen, Jian Zheng

**Affiliations:** 1 Radiology, Guangdong Medical University, Zhanjiang, CHN; 2 Ultrasound, The Third People's Hospital of Longgang District, Shenzhen, CHN; 3 Gynecology, The Third People's Hospital of Longgang District, Shenzhen, CHN; 4 Ultrasound, The Second Affiliated Hospital, School of Medicine, The Chinese University of Hong Kong, Shenzhen &amp; Longgang District People’s Hospital, Shenzhen, CHN

**Keywords:** cystic hyperplasia, endometrial cavity fluid, endometrial polyp, postmenopausal, ultrasound

## Abstract

Endometrial polyps, a common gynecological finding, may be symptomatic or asymptomatic, with age and postmenopausal bleeding identified as significant risk factors. While typically identifiable on ultrasound, atypical presentations can pose diagnostic challenges. We report a case of a postmenopausal woman initially misdiagnosed with endometrial cavity fluid due to the polyp's almost entirely cystic appearance on ultrasound. Hysteroscopy revealed hyperplastic endometrium with a 3.5-cm endometrial polyp. A polypectomy was performed, and the specimen was sent for histopathological evaluation, which revealed an endometrial polyp and cystic hyperplasia without atypia. This case highlights the importance of considering atypical sonographic presentations of endometrial polyps, particularly in postmenopausal women, to avoid diagnostic delays and guide appropriate management.

## Introduction

Endometrial polyps (EPs) are a frequent cause of abnormal uterine bleeding in premenopausal and postmenopausal women, although some cases remain asymptomatic. In women with EPs, symptomatic vaginal bleeding and postmenopausal status are identified as significant risk factors for endometrial malignancy [1]. Ultrasonography is the primary investigation for EP [2]. Typical EPs present sonographically as well-defined, homogeneous, polypoid lesions isoechoic to the endometrium, preserving the endometrial-myometrial interface. Atypical features may include cystic components, multiplicity, a broad base, and hypoechogenicity or heterogeneity [3]. These atypical polyps pose a significant challenge to ultrasound diagnosis. We present a case of an EP with an unusual, predominantly cystic sonographic appearance, which led to an initial misdiagnosis of endometrial fluid.

## Case presentation

**Figure 1:**
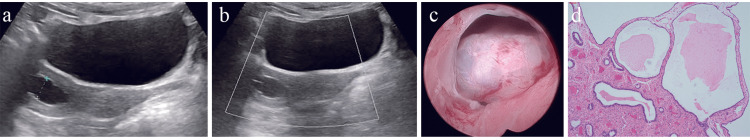
Grayscale ultrasound demonstrating an anechoic fluid collection within the uterine cavity (a). Color Doppler imaging reveals no flow signals within the fluid (b). Hysteroscopic finding of a large endometrial polyp (c) with microscopically demonstrated cystic changes without atypia (d).

A 66-year-old postmenopausal woman presented for a routine health checkup. She had no history of medication use, specifically hormone therapy. Physical examination, including pelvic examination, was unremarkable. Ultrasound revealed an endometrial cavity fluid collection measuring 1.4 cm, with anechoic fluid and no color (Figures 1a, 1b). Despite the seemingly benign sonographic appearance, her age raised clinical suspicion, and hysteroscopy with biopsy was recommended for definitive evaluation.

Hysteroscopy revealed a large, 3.5-cm EP within the uterine cavity (Figure 1c). The polyp was removed, and pathological examination confirmed the diagnosis of an EP, measuring 3.5 x 2.5 x 1 cm. Microscopically, the polyp demonstrated cystic changes without atypia (Figure 1d).

## Discussion

EPs are localized growths of endometrial tissue composed of varying amounts of glands, stroma, and blood vessels covered by epithelium. Established risk factors for EP development include advancing age, hyperestrogenism, hypertension, and tamoxifen use [4]. The prevalence is reported to be between 7.8% and 34.9% [5]. While EPs are usually benign, Savelli et al. reported a risk of malignancy of 0%-4.8% for these lesions [6], and Lee et al. found that postmenopausal women have an increased risk of endometrial malignancy [1]. Endometrial cancer usually manifests as a diffuse process; however, in early disease stages, it can occasionally present as a polypoid mass [3]. Consequently, timely diagnosis and treatment are crucial for appropriate management and minimizing the risk of complications.

Ultrasonography, particularly transvaginal ultrasound, is the primary investigation for EP [2], typically appearing as well-defined, homogeneous, polypoid, isoechoic lesions preserving the endometrial-myometrial interface. However, atypical features such as cystic components, multiplicity, a broad base, and heterogeneous or hypoechoic echotexture may be observed; indeed, polyps can occasionally present with a heterogeneous echotexture and multiple cysts [3]. This suggests hemorrhage, infarction, or inflammation within the polyp [7]. Such cystic polyps are more commonly observed in postmenopausal women, especially those without abnormal uterine bleeding [8,9]. In endometrial cystic lesions, cysts can present as regular or irregular in shape. Irregular cystic areas were seen predominantly in malignant lesions [10]. A polyp typically demonstrates a "feeding vessel" (single vessel with or without branching) on Doppler color imaging [9].

In our case, the patient's uterine cavity appeared almost entirely anechoic on ultrasound, a finding inconsistent with the typical ultrasound features of a polyp, such as a well-defined, homogeneous, hyperechoic lesion with regular contours and a feeding vessel. Furthermore, the ultrasound findings in our case differed significantly from the common cystic changes seen in polyps. While cystic changes within polyps generally manifest as singular or multiple small cystic areas within a polyp-like mass, our case presented with a completely cystic appearance on ultrasound, leading to an initial misdiagnosis of endometrial cavity fluid. However, during hysteroscopic surgery, a mass measuring about 3.5 cm was identified, which was ultimately confirmed to be a polyp by pathology.

## Conclusions

This case highlights the diagnostic challenges posed by EPs with atypical ultrasound presentations. While typically identifiable on ultrasound, the almost entirely cystic appearance of the polyp in this case led to an initial misdiagnosis. This case underscores the importance of considering atypical presentations of EPs, particularly in postmenopausal women, and highlights the value of further investigation with hysteroscopy when ultrasound findings are inconclusive.
